# A study of the relationship between the Wnt/β-catenin signaling pathway and the gastrointestinal development of rat embryonic and perinatal periods

**DOI:** 10.3892/etm.2013.1058

**Published:** 2013-04-09

**Authors:** BOTAO WEI, YONGSHENG GUO, JIA ZHAI, JUAN SU, LEI HAN, CHUNSHENG KANG, QINGYU ZHANG

**Affiliations:** 1 Department of Gastroenterology, Tianjin Medical University General Hospital; Tianjin 300052, P.R. China; 2 Department of Infectious Disease, Tianjin Children’s Hospital; Tianjin 300052, P.R. China; 3 Department of Neurosurgery, Tianjin Medical University General Hospital, Tianjin Neurological Institute, Key Laboratory of Post-trauma Neuro-repair and Regeneration in Central Nervous System, Ministry of Education, Tianjin Key Laboratory of Injuries, Variations and Regeneration of Nervous System, Tianjin 300052, P.R. China

**Keywords:** β-catenin, signal transduction, gastrointestinal development, rat

## Abstract

The Wnt/β-catenin signaling pathway plays a critical role in directing cell fate during the embryonic development of animals and humans. To investigate the effects of the Wnt/β-catenin signaling pathway on gastrointestinal development and differentiation, we studied the expression pattern of β-catenin, a key component of the pathway, in the gastrointestinal tissues of embryonic and perinatal rats. Immunohistochemistry was used to examine the expression levels of β-catenin in Sprague Dawley (SD) rat embryos at days 13, 18 and 21 and in SD rats at 1, 3, 7 and 28 days of age. We observed that the expression of β-catenin was greater and more diffuse in the gastrointestinal tissues of rat embryos at days 18 and 21 of gestation and in SD rats at days 1 and 3. In conclusion, our data suggest that β-catenin also plays an important role in the development of gastrointestinal tissues during the middle and late embryonic periods and the early postnatal period.

## Introduction

The Wnt gene family encodes evolutionarily conserved proteins that regulate processes in adults and in embryonic development (1–4). The Wnt signaling pathway impacts the development of the liver, pancreas, intestines, lungs and other endodermal organs, and the development of the mesodermal organs in early embryonic development by appropriate spatial and temporal mechanisms (5–9). Numerous WNT signaling pathways are involved in the signal transduction process and the canonical Wnt/β-catenin signaling pathway is the best characterized pathway in this process.

The Wnt/β-catenin signaling pathway is topic of great interest in biomedicine. Numerous studies have confirmed that the key activator of the Wnt/β-catenin signaling pathway is the nuclear translocation of β-catenin. As a key signaling protein, its accumulation in the cytoplasm and translocation into the nucleus mark the activation of this signaling pathway (10,11). However, few studies have addressed its role in the generation and development of gastrointestinal tissues. In the current study, we investigated the primitive guts of Sprague Dawley (SD) rats during the middle and late embryonic periods and the gastrointestinal tissues of rats during the perinatal period. Using immunohistochemical methods and a clear understanding of the normal developmental pattern from morphological and histological characterizations, we examined the temporal expression pattern of β-catenin in the Wnt/β-catenin signaling pathway in the gastrointestinal tissues of embryonic and adult rats. Furthermore, we discuss the role of the pathway in gastrointestinal development.

## Materials and methods

### Animals

SD rats (20 females and 10 males) were purchased from the animal center of the Cancer Institute of the Chinese Academy of Medical Sciences (Beijing, China), and bred at the facility of laboratory animals in Tianjin University (Tianjin, China). All experimental procedures were carried out according to the regulations and internal biosafety and bioethics guidelines of Tianjin Medical University and the Tianjin Municipal Science and Technology Commission (Tianjin, China). The rats were housed together each night and vaginal secretions were obtained the next morning. Day 0 of gestation was marked as the day when sperm was discovered. Since the average fertility cycle of rats is 28 days, the integrated embryos were removed by cesarean section from anesthetized female rats at days 13, 18 and 21 of gestation and then fixed in 4% paraformaldehyde. Additionally, the gastrointestinal tissues of rats at days 1, 3, 7 and 28 of age were removed and fixed in 4% paraformaldehyde.

### Immunohistochemistry

After the embryos and the gastrointestinal tissues of rats were fixed, paraffin-embedded tissue sections were used for the examination of β-catenin expression. The sections were dewaxed, treated with 3% H_2_O_2_ for 10 min and incubated with the appropriate antibody (1:200 dilutions; Signalway Antibody, College Park, MD, USA) overnight at 4°C. Biotinylated secondary antibody (1:200 dilutions; Signalway Antibody) was added at room temperature for 1 h, which was followed by incubation with ABC-peroxidase for an additional hour. After washing with Tris buffer, the sections were incubated with 3,3′-diaminobenzidine (DAB, 30 mg dissolved in 100 ml Tris buffer containing 0.03% H_2_O_2_) for 5 min, rinsed in water and counterstained with hematoxylin.

The average positive rates of β-catenin were determined by examining slices in each of 10 randomly selected fields that were chosen by the same observer. The β-catenin staining in the cytoplasm and the nucleus of each cell was analyzed according to Duncan *et al*(12).

### Statistical analysis

The statistical analysis was performed using SPSS 16.0 software (SPSS, Inc., Chicago, IL, USA). To compare the expression-positive rates of β-catenin at various time points, a χ^2^ test was applied to compare the numerical data among groups. P<0.05 was considered to indicate a statistically significant result.

## Results

### β-catenin expression in the cytoplasm and nuclei of gastric cells at various time points

**Figure 1:**
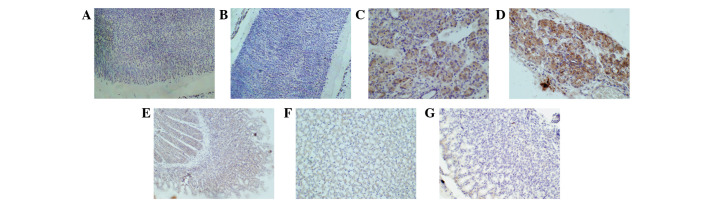
β-catenin expression in the cytoplasm and nuclei of gastric cells at various time points. (A–C) β-catenin in rat embryos at days 13, 18 and 21, respectively. (D–G) β-catenin in the gastric cells of SD rats at days 1, 3, 7 and 28 of age, respectively (immunohistochemical staining; magnification, ×100).

**Figure 2:**
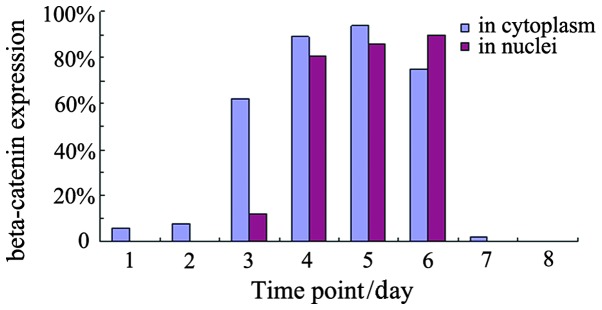
β-catenin expression in the cytoplasm and nuclei of gastric cells at various time points. Time points 1, 2, 3, 4, 5, 6 and 7 represent days 13, 18 and 21 of gestation and days 1, 3, 7 and 28 of age, respectively. Series 1, β-catenin expression in the cytoplasm of gastric cells at various time points, Series 2, β-catenin expression in the nuclei of gastric cells at various time points.

Fig. 1 shows the expression of β-catenin in gastric cells. First, β-catenin expression was observed in the cytoplasm at each of the selected time points and the expression-positive rates were 6% (day 13 of gestation), 8% (day 18 of gestation), 62% (day 21 of gestation), 89% (day 1 of age), 94% (day 3 of age), 75% (day 7 of age) and 2% (day 28 of age). After statistical comparisons were conducted between any two samples, the differences in the expression-positive rate among samples from days 13 and 18 of gestation and day 28 of age were not statistically significant (P>0.05) due to the low positive rates of these samples. However, comparing each of these data with data obtained from day 21 of gestation and days 1, 3 and 7 of age, respectively, the differences were statistically significant (χ^2^=408.73, P<0.001). The positive rates were higher in the latter samples. Secondly, in the nuclei of the samples taken at the 7 chosen time points, the expression of β-catenin was only observed in the rats at day 21 of gestation and days 1, 3 and 7 of age, which corresponded to positive rates of 12%, 81%, 86% and 90%, respectively. Statistical comparisons between any two samples showed that the differences between the sample at day 21 of gestation and the latter three samples were statistically significant (χ^2^=186.65, P<0.001), whereas the differences between the samples taken at days 1, 3 and 7 of age were not statistically significant (P>0.05). The positive rate of the sample taken at day 21 of gestation was low, whereas the rates of the latter three samples were high (Fig. 2).

### β-catenin expression in the cytoplasm and nuclei of intestinal cells at various time points

**Figure 3:**
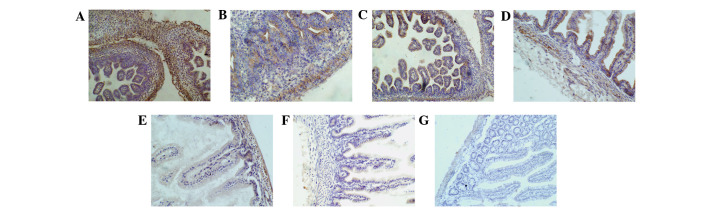
β-catenin expression in the cytoplasm and nuclei of intestinal cells at various time points. (A–C) β-catenin in rat embryos at days 13, 18 and 21, respectively. (D–G) β-catenin in the intestinal cells of SD rats at days 1, 3, 7 and 28, respectively (immunohistochemical staining, magnification, ×100).

**Figure 4:**
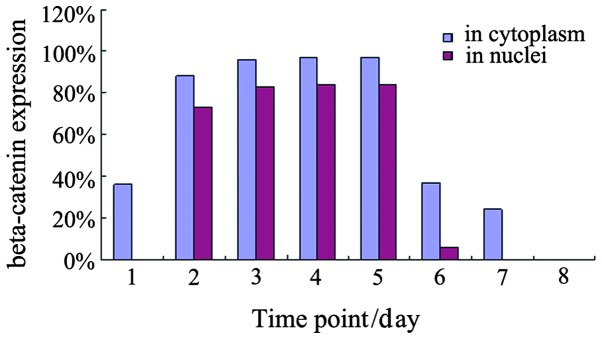
β-catenin expression in the cytoplasm and nuclei of intestinal cells at various time points. Series 1, β-catenin expression in the cytoplasm of intestinal cells at various time points. Series 2, β-catenin expression in the nuclei of intestinal cells at various time points. Time points 1, 2, 3, 4, 5, 6 and 7 represent days 13, 18 and 21 of gestation and days 1, 3, 7 and 28 of age, respectively.

Fig. 3 shows β-catenin expression in the cytoplasm and nuclei of intestinal cells. β-catenin expression was observed in the cytoplasm of cells taken at the 7 time points and the positive rates were 36% (day 13 of gestation), 88% (day 18 of gestation), 96% (day 21 of gestation), 97% (day 1 of age), 97% (day 3 of age), 37% (day 7 of age) and 24% (day 28 of age). After a statistical analysis of these data was conducted, the differences in the expression-positive rate among samples at day 13 of gestation and days 7 and 28 of age were not statistically significant since the positive rates were too low (P>0.05). However, when each of these samples was compared with samples taken at day 18 and 21 of gestation and days 1 and 13 of age, the differences were statistically significant (χ^2^=311.16, P<0.001). The positive rate was higher in the latter samples. β-catenin expression was also observed in the nuclei of intestinal samples taken from rats at days 18 and 21 of gestation and days 1, 3 and 7 of age. Using statistical analysis, we identified that the differences between the sample taken at day 7 of age and each of the other four samples were statistically significant (χ^2^=204.37, P<0.001), whereas the differences among days 18 and 21 of gestation and days 1 and 3 of age were not (P>0.05). The positive rates were low for the samples taken at day 7 of age, whereas the rates were high for the samples taken at the other four time points (Fig. 4).

### Expression pattern of β-catenin in various tissues and developmental phases

**Figure 5:**
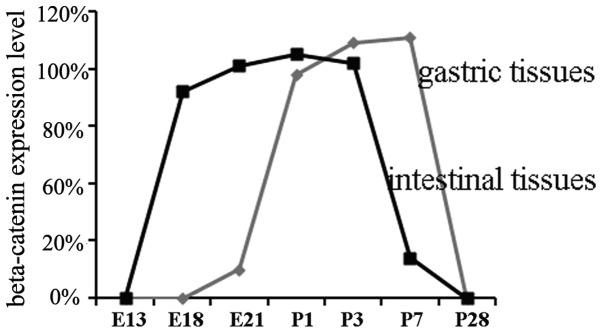
Comparison of the time points when β-catenin entered the nuclei of gastrointestinal cells and reached its peak expression level. E13, E18, E21, P1, P3, P7 and P28 represent days 13, 18 and 21 of gestation and days 1, 3, 7 and 28 of age, respectively.

In gastric cells, β-catenin had moved from the cytoplasm into the nuclei by day 21 of gestation. The expression reached a peak level at days 1, 3 and 7 of age, and then the expression began to decrease at day 7 of age. Within intestinal cells, β-catenin moved from the cytoplasm into the nuclei by day 18 of gestation, reached a peak level at day 21 of gestation and days 1 and 3 of age, and then began to decrease at day 3 of age. β-catenin moved from the cytoplasm into the nuclei and reached a peak level in intestinal tissues earlier than in gastric tissues. This time point corresponded to when the signal intensity began to decrease (Fig. 5).

## Discussion

Nusse *et al*(13) reported the first Wnt gene when the authors induced breast cancer into mice using the mouse mammary tumor virus (MMTV). Since this discovery in 1982, 19 types of human Wnt genes have been identified (14). The Wnt signaling pathway has also been shown to be divided into three main branches: i) the canonical Wnt/β-catenin signaling pathway. This pathway regulates the expression of target genes of the Wnt/β-catenin signaling pathway through the β-catenin/T-cell factor (TCF) (15,16). ii) The planar cell polarity (PCP) pathway. This pathway regulates cytoskeletal rearrangement within cells, establishes asymmetric cell polarity and coordinates changes in cell morphology and cell movement (17). iii) The Wnt/Ca^2+^ pathway. This pathway regulates cellular adhesion and viability (18). Research on Wnt signal transduction showed that β-catenin, a protein mediating cellular adhesion and cellular junctions, is crucial for the Wnt signaling pathway (19,20). Under normal conditions, the majority of β-catenin in the cytoplasm binds E-cadherin and cytoskeletal proteins to maintain the stability of cells. However, some β-catenin binds adenomatous polyposis coli (APC), glycogen synthase kinase 3β (GSK3β) and axin and then the ubiquitin-proteasome system through GSK3β phosphorylation and the β-transducin-repeat-containing protein (β-TrCP). β-TrCP degrades β-catenin and thereby maintains low levels of free β-catenin in the cytoplasm that prevent β-catenin from entering the nucleus to activate target genes (21). When the Wnt signaling pathway is activated, Wnt binds the receptors Frizzled (Fz) and LRP5/6 to activate the dishevelled protein (Dvl/Dsh) within cells. Dvl then inhibits the phosphorylation of β-catenin by GSK3β. The unphosphorylated β-catenin remains stable in the cytoplasm, accumulates gradually, moves into the nucleus and then binds transcription factors of the TCF/LEF family to control the transcription and expression of downstream target genes and fully promote the development of tissues.

A previous study suggests that Wnt/β-catenin plays an important role in the early development of the embryo (22). For example, at the two-cell stage of *Xenopus* development, β-catenin is distributed mainly in the cytoplasm of dorsal cells, whereas during the 16-cell stage to 32-cell stage; it is mainly distributed in the nuclei of dorsal cells. However, if the β-catenin in the rat embryo is knocked out, the development of the embryonic primary body axis fails. Few studies have systematically investigated whether β-catenin plays an important role during the middle and late embryonic periods and the early postnatal period.

In the longitudinal sections of gastric tissues from the primitive gut shown in Fig. 1A and B, we observed a few light brown stains in the cytoplasm of the mucousal, submucousal, muscular, and serous layers; these stains showed that β-catenin exists in the cytoplasm of gastric cells at these two time points, but it was small in quantity and weak in signal expression. In Fig. 1C–E, we observed stronger brown stains in gastric cells, and these stains were observed in the cytoplasm and in the nuclei. The stains gradually became more evident, and the signal expression increased. This result showed that β-catenin had moved from the cytoplasm into the nucleus by day 21 of gestation, which indicated that the β-catenin signaling pathway was activated at this time point. This movement had reached a peak in the rats at days 1 and 3 of age. In Fig. 1F, the positive rates of brown stains in the gastric cytoplasm and nuclei of the rats at day 7 of age remained at the peak level, but the color was lighter. In Fig. 1G, the brown stains in the cytoplasm and nuclei of the rat gastric cells at day 28 of age had almost disappeared. This result showed that β-catenin expression had weakened in the gastric cells of the rat at day 7 of age, and a small amount of β-catenin existed in the cytoplasm of rat gastric cells at day 28 of age when gastric structures are formed (including the gastric lamina propia and gland, shown in Fig. 1G). Similarly, in the primitive rat gut at day 13 of gestation, shown in Fig. 3A–G, the brown stains were not evident in the mucousal and submucousal layers; these stains were clearly visible in the muscular layer and adventitial cells, which showed that β-catenin had accumulated in the cytoplasm. The brown stains were observed in the cytoplasm and nuclei of rat intestinal cells at days 18 and 21 of gestation and days 1, 3 and 7 of age. At day 21 of gestation and days 1 and 3 of age, the positive rates of these samples gradually increased to a peak, but the stain intensity decreased at day 3 of age. These data suggest that β-catenin moved from the cytoplasm into the nuclei at day 18 of gestation and that the β-catenin signaling pathway was most strongly activated at day 21 of gestation and days 1 and 3 of age but then decreased after day 3 of age. The brown-stain positive rates and stain intensity were markedly lower in the cytoplasm and nuclei of the rat intestinal cells at day 7 of age, and the brown stains disappeared from the nuclei at day 18 of age. This finding indicated that a small amount of β-catenin existed in the cytoplasm of the rat intestinal cells at day 28 of age. However, Fig. 3G showed the intestinal structure, including the intestinal villi and glands.

In summary, we examined the development and differentiation of gastrointestinal tissues in the middle and late embryonic periods and the early postnatal period. We observed that the expression of β-catenin in the gastrointestinal tissues was present at day 13 of gestation, activated from day 18 of gestation to day 3 of age, and began to decrease at day 7 of age. These data suggest that β-catenin plays an important role by promoting the development of gastrointestinal tissues in the middle and late embryonic periods and the early postnatal period. However, after tissues and organs mature, β-catenin is maintained at a low level in the cytoplasm so it is not able to promote development. The results of these experiments suggest that β-catenin plays an important role in gastrointestinal tissue development during the middle and late embryonic periods and the early postnatal period, which provides the necessary experimental evidence for further studies concerning the mechanism of this signaling pathway, tumorigenesis and tumor treatment.
